# Maternal Inheritance of *Twist* and Analysis of MAPK Activation in Embryos of the Polychaete Annelid *Platynereis dumerilii*

**DOI:** 10.1371/journal.pone.0096702

**Published:** 2014-05-02

**Authors:** Kathrin Pfeifer, Christoph Schaub, Katrin Domsch, Adriaan Dorresteijn, Georg Wolfstetter

**Affiliations:** Institut für Allgemeine und Spezielle Zoologie; Allgemeine Zoologie und Entwicklungsbiologie, Justus-Liebig-Universität Gieβen, Gieβen, Germany; University of North Carolina at Chapel Hill, United States of America

## Abstract

In this study, we aimed to identify molecular mechanisms involved in the specification of the 4d (mesentoblast) lineage in *Platynereis dumerilii*. We employ RT-PCR and *in situ* hybridization against the *Platynereis dumerilii twist* homolog (*Pdu-twist*) to reveal mesodermal specification within this lineage. We show that *Pdu-twist* mRNA is already maternally distributed. After fertilization, ooplasmatic segregation leads to relocation of *Pdu-twist* transcripts into the somatoblast (2d) lineage and 4d, indicating that the maternal component of *Pdu-twist* might be an important prerequisite for further mesoderm specification but does not represent a defining characteristic of the mesentoblast. However, after the primordial germ cells have separated from the 4d lineage, zygotic transcription of *Pdu-twist* is exclusively observed in the myogenic progenitors, suggesting that mesodermal specification occurs after the 4d stage. Previous studies on spiral cleaving embryos revealed a spatio-temporal correlation between the 4d lineage and the activity of an embryonic organizer that is capable to induce the developmental fates of certain micromeres. This has raised the question if specification of the 4d lineage could be connected to the organizer activity. Therefore, we aimed to reveal the existence of such a proposed conserved organizer in *Platynereis* employing antibody staining against dpERK. In contrast to former observations in other spiralian embryos, activation of MAPK signaling during 2d and 4d formation cannot be detected which questions the existence of a conserved connection between organizer function and specification of the 4d lineage. However, our experiments unveil robust MAPK activation in the prospective nephroblasts as well as in the macromeres and some micromeres at the blastopore in gastrulating embryos. Inhibition of MAPK activation leads to larvae with a shortened body axis, defects in trunk muscle spreading and improper nervous system condensation, indicating a critical function for MAPK signaling for the reorganization of embryonic tissues during the gastrulation process.

## Introduction

Early development in the marine polychaete annelid *Platynereis dumerilii* follows a canonical spiral cleavage mode leading to blastomeres with distinct volumes and cytoplasmatic compositions [1], [2]. Upon fertilization, a cytoplasmatic movement termed ooplasmatic segregation induces a flow of clear cytoplasm from the center of the zygote towards the future animal pole. Simultaneously, yolk granules and lipid droplets re-arrange towards the vegetal pole of the fertilized egg [1], [3], [4]. Following an invariant unequal cleavage pattern, the majority of the clear cytoplasm is distributed into the largest blastomere at the four-cell stage, the so-called D-blastomere. Later in development, the D-blastomere will give rise to the D-quadrant including the somatoblast (2d micromere) and mesentoblast (4d micromere) that represent the progenitors of most trunk-forming cells in *Platynereis*. Both cells receive remarkably high amounts of clear cytoplasm which makes them distinguishable from all other, rather yolky blastomeres [1]. Altering the regular cytoplasmatic composition of the early blastomeres or provoking equalized cleavages by experimental manipulation leads to characteristic Janus malformations of the trunk suggesting that the distribution of early morphogenetic determinants is crucial for later trunk and axis formation [5]–[7]. However, these early molecular determinants decisive in the specification of lineage fates are still unknown in *Platynereis*.

Recently, a single *Platynereis twist* ortholog has been identified and robust *Pdu-twist* expression was observed in the developing larval trunk musculature [8]. Since the trunk mesoderm can be traced back to the 4d blastomere [9] we aimed to analyze the mechanisms involved in the fate specification of this cell. Therefore, we employed *Pdu-twist* expression as a marker to follow the development of the early 4d lineage. Interestingly, *Pdu-twist* transcripts are maternal contributions to the oocyte and the fertilized egg where they subsequently become selectively distributed to the 2d and 4d lineages during ooplasmatic segregation and the subsequent cleavages. However, selective enrichment of *Pdu-twist* mRNA in 4d itself is not observed, but occurs in the myogenic descendants after the separation of the germ line from the mesendodermal lineage is completed.

Experimental studies in the mud snail *Ilyanassa* revealed a conserved connection between mesoderm specification and the activity of an ‘embryonic organizer’ functionally linked by activation of the mitogen-activated protein kinase/extracellular signal-regulated kinase (MAPK/ERK) signaling pathway. Since MAPK activation has been observed in certain blastomeres of the D-quadrant in four other mollusc species and in the 4d micromere of the sedentary polychaete *Hydroides hexagonus* it has been tempting to speculate about a conserved role for the embryonic organizer in the specification of the mesodermal lineage or even 4d [10]–[14]. However, a recent analysis by Amiel et al. (2013) reports the absence of MAPK activation during the early development of *Capitella sp*.I indicating a high level of variability even within the polychaete annelids [15]. To reveal the existence of a proposed organizer in *Platynereis* we employed antibody staining against di-phosphorylated MAPK/ERK. Analyzing MAPK activation in *Platynereis*, we could not observe ERK phosphorylation during early embryonic development including formation of the 2d and 4d blastomeres which would not support the former assumption of a conserved connection between mesoderm induction and organizer function involving MAPK signaling. Since our observations revealed ERK phosphorylation in macromeres and some micromeres around the blastopore at the onset of gastrulation we wished to analyze the functional role of MAPK signaling during this process. Abrogation of ERK phosphorylation employing the MAPK/ERK kinase (MEK) U0126 inhibitor leads to severe defects in larval muscle organization and nervous system condensation, thus revealing requirement of MAPK signaling for the rearrangement of embryonic tissues.

## Material and Methods

### Ethics statement

All animal work was conducted according to the national and European guidelines for animal research.

### *Platynereis dumerilii* culture

Standard *Platynereis dumerilii* culture methods were followed [16].

### Developmental RT-PCR Analysis

Total RNA from different developmental stages was isolated (RNeasy, Qiagen), DNaseI (Sigma) treated, and cDNA was synthesized from 1 µg RNA using Omniscript RT Kit (Qiagen) with Poly-dT_10–20_ (Qiagen). Primers used were: CGC AAC TCA GAA AGA TCA TCC and TTC AAG ACC GCT TGA CTG AA (*Pdu-twist*) and AGA TCT GGC ATC ACA CCT TCT AC and CTC GTG GAT ACC AGC GGA TTC (*Pdu-actin*). Amplification parameters were: 5 minutes 94°C, 35× (45 seconds 94°C, 45 seconds 58°C, 1 minute 72°C) followed by 10 minutes at 72°C. For negative controls, either reverse transcriptase or template was omitted.

### Northern analysis

Total RNA from different developmental stages (10 µg per lane) was subjected to northern analysis according to standard procedures. Digoxigenin labeled antisense *Pdu-twist* RNA probes (10 ng/ml) were hybridized over night at 65°C. Chemoluminescent detection was performed with alkaline phosphatase coupled anti-Digoxigenin antibodies (Roche) and CSPD (Roche) as substrate. Chemoluminescence was recorded on X-ray film (Kodak) and developed.

### Cloning of *Pdu-Myosin heavy chain*

A fragment of *Platynereis dumerilii Myosin heavy chain (Pdu-Mhc)* was amplified in a PCR-reaction (supplemented with 3 mmol MgCl_2_) on total 48 h cDNA library with the following degenerated primers: GCA ACG CCA AGA CCG TG(AC) G(AGCT) AA(CT)(AG)A(CT)AA and CGA TGC CCT CCT TCT TGT ACT C(CT) TC(CT) TG(CT) TC. The complete 5′ cDNA region of *Pdu-Mhc* was isolated by semi-nested rapid amplification of cDNA ends (RACE) with the BD Smart™ RACE cDNA Amplification Kit (BD Biosciences Clontech). We used the gene-specific primers GCT GCA GAC GCT CGT TGG TGT AGT TGA TGC AAG GCT GC, and CTG TCG TAG AGG GAC TTG GCC AGG GCA GCC AC in combination with the universal and semi-nested universal primers provided by the Smart™ RACE kit. The amplified fragments were subsequently cloned into the pGEMT-Easy (Promega) vector and confirmed by sequencing (Seqlab). The Pdu-Mhc 5′cDNA sequence has been uploaded to the National Center for Biotechnology Information (NCBI) nucleotide sequence database (http://www.ncbi.nlm.nih.gov/).

### Preparation and fixation of embryos

The egg jelly surrounding the embryos was removed by several washes with natural sea water (NSW) over a 75 µM mesh gaze. Prior to fixation, embryos from 0.5 hours post fertilization (hpf) up to 14.5 hpf were treated for 2×4 min with 50 mM Tris supplemented with 495 mM NaCl, 9.6 mM KCl, 27.6 mM Na_2_SO_4_, 2.3 mM NaHCO_3_ and 6.4 mM EDTA (pH 8.0) to permeabilize the vitellin envelope (R. Kostyuchenko personal communication, also referred to as TCMFSW by Schneider and Bowerman) [17]. Fixation was performed overnight in phosphate buffered saline (PBS, pH 9.5) containing 3.7% formaldehyde at 4°C. After fixation, specimens were washed several times in PTw (PBS containing 0.1% Tween-20). Embryos older than 15 hpf were digested for 5 min with 10 µg/ml Proteinase K in PTw while Proteinase treatment for younger stages was omitted. Digestion was stopped by washing the embryos for 2×5 min in PTw containing 2 mg/ml glycine.

### Whole mount *in situ* hybridization (WMISH)

Sequence information about the *Pdu-twist* probes, and a WMISH protocol are described in Pfeifer et al. [8]. *Pdu-Mhc* probes were generated from a PCR product (primer sequences: CCT CAT GGG AGC AAC GC and GGG CCT CGA ACT TTG CAT TC) of the *Pdu-Mhc* full-length clone (see above) that was sub-cloned into the pGEMTEasy vector (Promega). Probes were synthesized using the Sp6- and T7-Megascript Kits (Ambion/Lifescience) and applied to the samples in a 0.5 ng/µl working concentration.

### Fuchsin staining

After WMISH, samples were stepwise dehydrated in a graded ethanol/ddH_2_O series (50%, 70%, 80%, 90%, 96%, 98% and 2×100% v/v ethanol respectively). Dehydrated embryos were re-fixed in a 20∶2.5∶1 mixture of 95% ethanol, 100% acetic acid and 37% formaldehyde for 1 h. Subsequently, specimens were washed 4×10 min in 70% ethanol and further treated with 2 M HCl for 10 min at 65°C. Embryos were washed once in ddH_2_O and 2 times in 70% ethanol. Afterwards, specimens were stained for 30 min with 5 mg/ml fuchsin basic (Carl Roth) dissolved in a 100∶1 mixture of 80% ethanol and 37% HCl. Embryos were rinsed at least 8 times in 100% ethanol and mounted in Euparal (Carl Roth).

### Antibody staining

Specimens were pre-incubated for 1 h in 1× blocking buffer (Roche Blocking Reagent diluted in 10 mM Maleic acid buffer pH 7.5 and supplemented with 15 mM NaCl). Samples were incubated on a shaker with the primary antibody at room temperature for 2 h and subsequently over night at 10°C. After several washes in PT (PBS containing 0.1% Triton X 100), samples were incubated overnight with the secondary antibody and FITC-conjugated Phalloidin (Sigma-Aldrich) in PT shaking at 10°C. Specimens were rinsed once and washed 4×20 min in PT at RT. Hoechst 33342 (Sigma-Aldrich) was applied in a dilution of 0.5 µg/ml in PT for 20 min whilst shaking at RT. Samples were mounted in FluoromountG (Southern Biotech) and analyzed under a Leica TCS SP2 confocal microscope. The following primary antibodies were used: rabbit anti Phospho-p44/42 MAPK (Erk1/2) (Thr202/Tyr204) Antibody (Cell Signaling #9101, dilution 1∶10 in Blocking buffer) and a mouse monoclonal antibody against acetylated tubulin (aat; Sigma-Aldrich T6793, applied dilution 1∶250 in PT). For secondary antibodies we employed Chromeo546-coupled anti-rabbit IgG (Abcam, dilution 1∶1500 in PT) and Cy3-conjugated anti-mouse IgG (Dianova, dilution 1∶200 in PT). Tyramide signal amplification (TSA, Life Technologies) was performed according to the manufactures instructions.

### Inhibition of MAPK activation

Embryos were incubated between 13.5 hpf and 16.5 hpf with various concentrations (10 µM, 25 µM or 50 µM) of the MEK Inhibitor U0126 (Promega) diluted in NSW. Dilutions were made from a DMSO stock solution containing 40 mM U0126. The total amount of DMSO in the incubation solution was adjusted to 0.5% in all experiments. Control embryos were taken from the same batch but cultured in pasteurized natural seawater (NSW) and treated with 0.5% DMSO in NSW for the same time period.

### Statistical analysis

Statistics were performed with the *STATISTICA 10* software package (StatSoft Inc.). Univariate Test of Significance for Percent and Tukey honest significant difference (HSD) post hoc analysis for multiple comparisons with a significance level of 0.05 was employed to analyze the distribution of four defined morphology classes within the samples. The analyzed dataset consisted of three individual experimental replicates for controls and inhibitor treatments, respectively. Total number of individuals analyzed: NSW (control): n = 913; 0.5% DMSO (vehicle control): n = 1545; U0126 (treatment): 10 µM, n = 1387; 25 µM, n = 1351; 50 µM, n = 1503.

## Results

### *Platynereis twist* mRNA is maternally deposited and specifically distributed into distinct embryonic lineages during early cleavages

**Figure 1:**
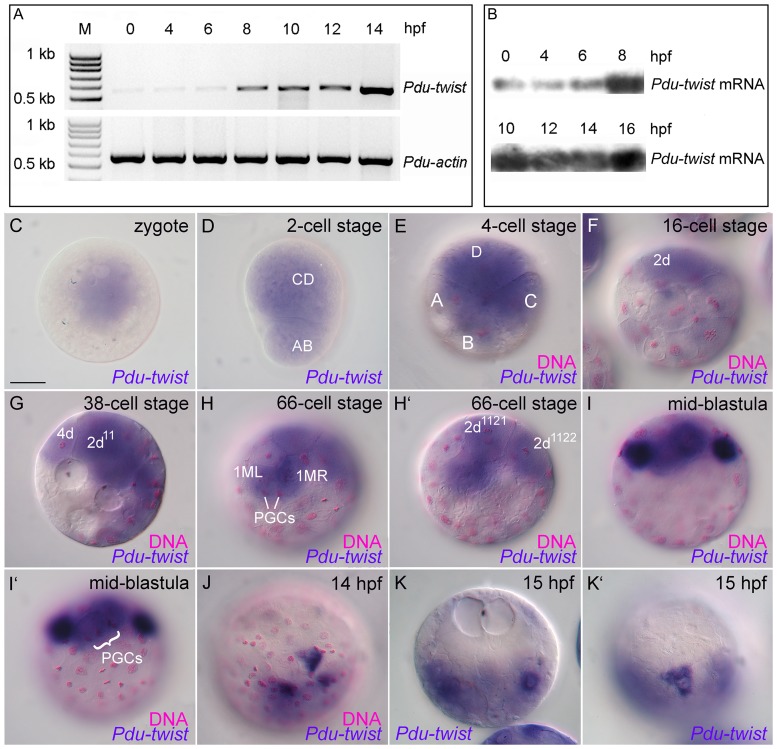
Detection and distribution of *Pdu-twist* mRNA during early embryonic development. **A, B.** Developmental RT-PCR (A) and Northern Blot (B) analyses reveal a maternal contribution of the *Pdu-twist* transcript. **C–K′.** Whole mount *in situ* hybridization against *Pdu-twist* (blue) counterstained with fuchsin (magenta in **C–J**) to reveal the positions of the nuclei. Maternal *Pdu-twist* transcript is detectable in the clear cytoplasm of the zygote (C). Ooplasmatic segregation and progressive cleavages displace maternal *Pdu-twist* mRNA into the CD blastomere (D), the D blastomere (E), and further into the somatoblast 2d (F), the mesentoblast 4d (G), and their early descendants (G–H). **I–I′.** Increased *Pdu-twist* expression can be observed within two cells of the 4d lineage after the separation of the primordial germ cells (PGCs) at 10 hpf. Different focal planes of the same embryo are shown to reveal *Pdu-twist* expression domains and PGC positions respectively. **J.** Robust *Pdu-twist* expression is detectable in three constricted cells at the blastopore around 14 hpf. **K–K′.** *Pdu-twist* expressing cells are detectable under the ectodermal cell layer and on the embryonic surface at 15 hpf. Blastomere staging was done according to Dorresteijn [1], D-lineage nomenclature after Henry et al. [20]. Scale bar: 50 µm. M =  Molecular weight marker (DNA), hpf  =  hours post fertilization.

To study the temporal transcription profile of *Platynereis twist* we performed developmental RT-PCR and Northern blot analyses. Both analyses uncovered a maternal component in the expression pattern of *Pdu-twist*. In the unfertilized zygote and prior to the start of the zygotic transcription, maternally distributed amounts of *Pdu-twist* mRNA can be detected in all investigated developmental stages (Fig. 1A, B). After 8 hours post fertilization (hpf) the level of *Pdu-twist* transcripts increases dramatically most likely indicating the start of zygotic transcription (Fig. 1A, B). To analyze the spatial distribution of *Pdu-twist* during these early developmental stages, we followed its localization by performing *whole mount in situ* hybridization (WMISH). A *Pdu-twist* sense control probe produced no detectable staining, revealing specifity of the signal generated by the antisense probe (suppl. Fig. S1A and B). Furthermore, sense- as well as antisense-probes of the much later expressed gene *Pdu-Pax6* [18] produced no staining in early stage embryos (data not shown). Our *in situ* hybridization results reveal that the maternally provided *Pdu-twist* mRNA is localized in a spatially restricted pattern within the zygote (Fig. 1C). In particular, the mRNA can only be found in the clear animal cytoplasm (Fig. 1C). After the first unequal cleavage, the majority of the *Pdu-twist* mRNA and the clear cytoplasm are distributed into the CD-blastomere (Fig. 1D), while the second unequal medial cleavage results in accumulation of the majority of the maternally inherited *Pdu-twist* transcripts in the largest of the four blastomeres, the D-blastomere (Fig. 1E). After the fourth cleavage, the somatoblast 2d has formed (Fig. 1F) [1], [9]. This is the largest of the micromeres in *Platynereis* and contains the highest amount of clear cytoplasm and *Pdu-twist* mRNA when compared to the other micromeres (Fig. 1F). After the sixth cleavage, the mesentoblast 4d is formed by the D-quadrant (Fig. 1G). Compared to 2d, 4d is a smaller micromere but receives a high amount of the clear cytoplasm [1], and contains maternal derived *Pdu-twist* transcripts (Fig. 1G). Further cleavages divide 4d in a bilateral pattern resulting in the two descendants 1ML and 1MR (also referred to as 4d^11^ and 4d^21^) at the 66 cell stage (Fig. 1H) [1], [17], [19], [20]. Both cells contain equal amounts of the *Pdu-twist* transcripts (Fig. 1H). Around the same time, the cells 2d^1121^ and 2d^1122^ are produced within the 2d lineage also by a bilaterally symmetrical division of 2d^112^ [1]. Interestingly, both blastomeres also contain *Pdu-twist* mRNA (Fig. 1H′). In the following two cleavage cycles, asymmetrical divisions of 1ML and 1MR and their larger progenies give, amongst others, rise to the primordial germ cells (PGCs in Fig. 1H) [1], [17], [19], [21], [22]. Interestingly, we could not detect any obvious differences in expression levels between the different embryonic lineages that inherit *Pdu-twist* transcripts up to this stage. Hence, apart from the quantities, there is no qualitative difference in maternally distributed *Pdu-twist* mRNA content between the mesentoblast (4d) and its early descendants and the blastomeres of the somatoblast (2d) lineage. However, after initiation of the zygotic transcription and after the separation of the smaller germ line precursors, we detected a strong increase in *Pdu-twist* mRNA expression in two of the larger descendants of ML and MR, while the other micromeres of the D-quadrant exhibit lower expression levels (Fig. 1I and I′). At 14 hpf, *Pdu-twist* expression is detectable in three superficial cells at the vegetal region of the blastopore (Fig. 1J). These blastomeres exhibit an intriguing triangular-shaped morphology with the sharp end pointing towards the blastopore (Fig. 1J). At 15 hpf, two highly *Pdu-twist* expressing cells on each side of the embryo are detectable, but when compared to stages of 14 hpf, the cells are now located underneath the surface and display a rounded morphology (Fig. 1K), indicating that they have been internalized by the epibolic gastrulation movements. However, one triangular-shaped *Pdu-twist* positive cell remains on the embryo surface (Fig. 1K′).

Taken together, our analysis reveals that *Pdu-twist* mRNA is contributed maternally during *Platynereis dumerilii* oogenesis. After fertilization of the oocyte, ooplasmatic segregation distributes these transcripts at the animal pole in zygotes. Later on, most of the maternally inherited *Pdu-twist* transcripts are selectively delivered into the D-blastomere and further into the somatoblast (2d) and mesentoblast (4d) lineages. Interestingly, we observed no selective enrichment of *Pdu-twist* mRNA at the 4d stage in particular blastomeres or differences in its distribution between the 2d and mesentoblast lineages. However, a strong increase in *Pdu-twist* levels are observed upon zygotic gene expression in descendants of the 4d lineage that then become internalized during later stages of the gastrulation process.

### Spatial and temporal analysis of MAPK activation during early *Platynereis* development

**Figure 2:**
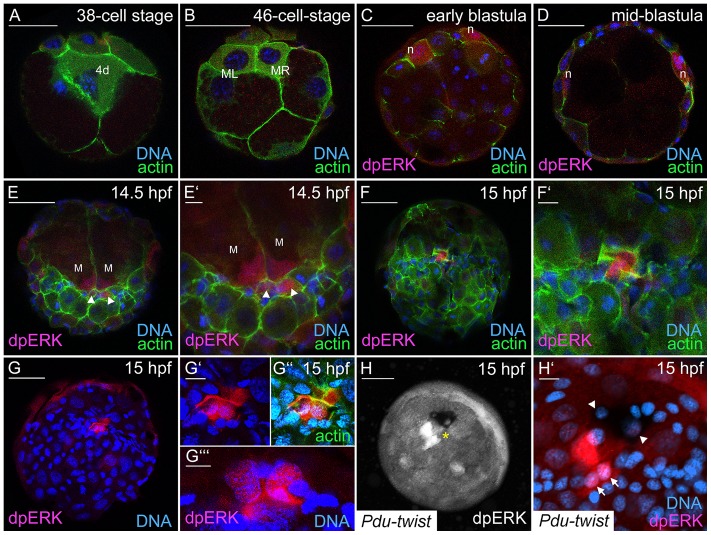
MAPK activation during early development in *Platynereis dumerilii*. Antibody staining against di-phosphorylated, activated MAPK/ERK (dpERK) in red or white, DNA-staining with Hoechst appears in blue, actin was marked by FITC-coupled Phalloidin (in green). **A, B** Embryos at the 38- and the 46-cell stage exhibit no dpERK staining in the mesentoblast (4d) and its descendants ML and MR. **C.** Initial dpERK staining was detected within the nephroblasts (n) in the animal hemisphere of a 7.5 hpf early blastula. **D.** MAPK activation is still visible during further head kidney development in the mid-blastula (10.5 hpf). **E–G″.** dpERK staining is visible during gastrulation in nuclei of small cells (arrowheads) and macromeres (M) in the region of the blastopore. **F–F′.** Micromeres with MAPK activity show an accumulation of filamentous actin at 15 hpf. **G′–G″.** MAPK positive cells in the region of the blastopore at 15 hpf. **G″′.** dpERK positive macromere nuclei in the same embryo as in G but different focal plane. **H, H′.** *Pdu-twist in situ* hybridization in combination with dpERK staining in a 15 hpf embryo. Activated MAPK and *Pdu-twist* positive cells are in close proximity at the region of the blastopore (asterisk). Arrows point towards two dpERK-positive nuclei that are in the same focal plane as the nuclei (arrowheads) of two *Pdu-twist* (black) expressing cells. Scale bars are 50 µm and 10 µm in whole embryo views and close-ups, respectively.

In *Ilyanassa*, the 4d blastomere is the result of a cell division that is accompanied by the activation of MAPK signaling. Moreover, MEK inhibition results in shortened larvae that lack a secondary body axis and several tissues derived from 4d, thus, intimately linking embryonic organizer activity to the specification of the mesentoblast (4d) lineage [13], [14]. To investigate MAPK activation during *Platynereis* development we detected MAPK/ERK di-phosphorylation with a phospho-p44/42 specific antibody (referred to as dpERK; Fig. 2, secondary antibody controls shown in Fig. S1C–D**′**). Since a complete annotation of the *Platynereis dumerilii* genome has not been reported we cannot exclude the possibility that the respective phosphorylation mechanism might not be conserved. However, the characteristic Threonine/Glutamic Acid/Tyrosine (TEY) phosphorylation motive and the activating loop of mammalian ERK1/2 proteins [23] are highly conserved from yeast to human and can be found in hypothetical protein sequences (GenBank: ELU13684.1, ESO03031.1 and ESO97339.1) derived from the recent genome annotations of three spiralian taxa [24]. Moreover, the sequence of a *Platynereis dumerilii* MAP kinase kinase homolog has been reported [25] (GenBank: CAJ38794.1), indicating that MAPK activation by MEK is conserved.

Staining for dpERK in *Platynereis*, we could not detect MAPK activation in the 2d and 3D blastomeres (data not shown), and the mesentoblast (38-cell stage; Fig. 2A). Moreover, MAPK activation was also absent in the 4d descendants, ML and MR (46-cell stage; Fig. 2B), indicating that this molecular mechanism is not active in these cells. The first activation of MAPK during embryonic development could be detected within two cells at the dorsal side within the animal hemisphere of the stereoblastula (Fig. 2C). These were identified as the embryonic nephroblasts [26], since a fluorescein tyramide substrate strongly precipitates in these cells and the later elongated head kidneys (Fig. S1E and F). The dpERK signal is still detectable in the mid-blastula (Fig. 2D) and within the elongating nephroblasts at 14.5 hpf (Fig. S1F) but decreases soon afterwards. During gastrulation, MAPK activation is detectable in 2–4 small cells in the blastopore region (14.5 hpf and 15 hpf; Fig. 2E–G″′). Interestingly, we observed that these cells exhibit an accumulation of filamentous actin and an elongated shape (Fig. 2F–F′ and G″). MAPK activation was also present within the macromeres (M, Fig. 2E–E′ and G″′) that stayed in close contact with the smaller dpERK-positive cells (Fig. 2E–E″). Double labeling for *Pdu-twist* mRNA and dpERK did not reveal MAPK activity in high *Pdu-twist* expressing cells at 15 hpf. However, triangular *Pdu-twist* expressing cells, and dpERK-positive cells are positioned in direct proximity to the blastopore (Fig. 2H–H′).

Taken together, we could not detect MAPK activation prior to and immediately after the production of the blastomeres 2d, 4d and their early descendants. The first cells in which we observed MAPK activation were the nephroblasts that give rise to the bilateral larval head kidneys. At the onset of gastrulation, we were able to show MAPK activation in micromeres around the blastopore and adjacent macromeres.

### Inhibition of MAPK activation causes muscle pattern defects and improper nervous system formation

**Figure 3:**
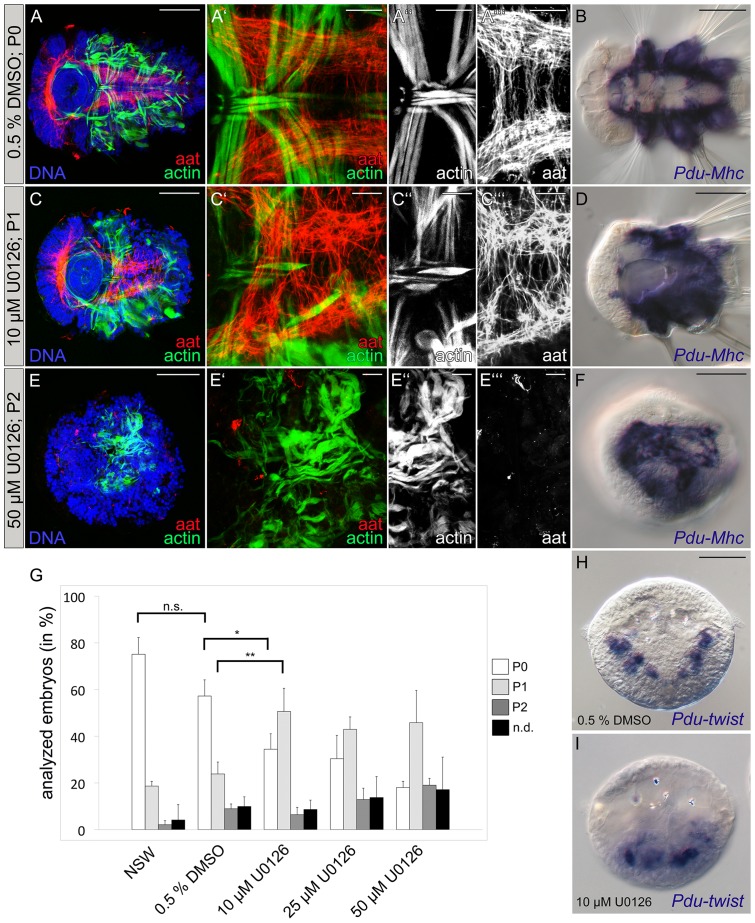
Inhibition of MAPK activity causes defects in muscle and nervous system development. Embryos were treated with 10 µM, 25 µM or 50 µM of the MEK inhibitor U0126. Control groups were incubated in 0.5% DMSO/NSW or pasteurized NSW from 13.5 to 16.5 hpf. All treated embryos from a clutch were collected and fixed 66 hpf for further analysis. **A–A″′, C–C″′, E–E″′.** Musculature of larvae was labeled with FITC-Phalloidin (actin in green or white), the nervous system was stained with an antibody against acetylated tubulin (aat in red or white), Hoechst labeling of DNA appears blue. **B, D** and **F** represent *Pdu-Mhc* detections (blue) after *in situ* hybridization. **A–A″′.** Normally developed muscles and nerves in a 66 hpf larvae of the 0.5% DMSO control group were classified as phenotype 0 (P0). **B.** *Pdu-Mhc* expression in a P0 larva at 66 hpf. **C–C’’’.** Larvae with a shortened body axis and reduced parapodial development were classified as phenotype 1 (P1). Muscle pattern defects occur due to abnormal positioned and orientated muscles. The nervous system is formed, but nerve fibers are messily arranged. **D.** *Pdu-Mhc* expression in a P1 larva. **E–E’’’.** Larvae classified as phenotype 2 (P2) lack a secondary body axis, fail to elongate and appear rounded in shape. Muscle accumulation is clearly observed and fibers of the ventral nervous system are missing. **F.** *Pdu-Mhc* expression in a P2 larva. **G.** Quantification of the proportions of non-developed eggs (n.d.), affected (phenotypes 1&2) and unaffected (phenotype 0) larvae in treatment and control groups. Means and standard errors of means are shown. Significance levels revealed by the Tukey HSD post hoc test are indicated for selected groups (*  = p<0.05, **  = p<0.01, n.s.  =  not significant/p>0.05). Data were obtained from three experimental replicates. Total Numbers (n) of counted larvae: NSW: n = 913, 0.5% DMSO: n = 1545, 10 µM: n = 1387, 25 µM: n = 1351 and 50 µM: n = 1503. **H–I.** *Pdu-twist* expression in control (H) and U0126 treated larva (I) at 24 hpf. Improper positioning of *Pdu-twist* expressing cells was observed in 45 of 310 larvae after 0.5% DMSO vehicle treatment and 161 of 270 larvae after treatment with 10 µM U0126. Scale bars are 50 µm and 10 µm for whole embryos and close ups, respectively.

Although our results did not reveal a role of MAPK signaling for organizer formation or 4d specification, dpERK staining was very prominent in cells at the blastopore region during the gastrulation stage. To further analyze a possible role of MAPK activation during the gastrulation process in *Platynereis*, we blocked MAPK/ERK phosphorylation employing the MAPK/ERK kinase (MEK) inhibitor U0126 [27], [28]. The inhibitor was applied 13.5 hpf, one hour prior to the observed MAPK activation in cells around the blastopore in the developing embryo (Fig. 2E), with either 10 µM, 25 µM or 50 µM U0126 and the treatment was continued until 16.5 hpf. After treatment, embryos were cultured in fresh NSW until 66 hpf. Inhibition of MAPK activation by U0126 was monitored by dpERK antibody staining after 1h treatment (Fig. S1G). To address possible side-effects of the general experimental handling and DMSO vehicle treatment, controls were performed by incubating batches of eggs in NSW or 0.5% DMSO/NSW. In order to avoid artificial selection and false negative results we did not collect swimming larvae due to their positive phototaxis but analyzed whole clutches of eggs that were fixed and stained after 66 hpf. Staining with FITC-coupled phalloidin and antibodies against acetylated tubulin (aat) was employed to visualize muscle- and nervous system-morphology (Fig. 3A–A″′, C–C″′, E–E″′). Additionally, we employed *in situ* hybridization against *Myosin heavy chain* (*Pdu-Mhc*) to analyze differentiation of the trunk muscles (Fig. 3B, D and F). Due to the experimental setup, we expected to observe also abnormally developed embryos within our samples that occasionally occur in laboratory cultures or might be a result of the general experimental procedures applied. This was confirmed on analysis of NSW and DMSO control clutches (Fig. 3G) that consisted mainly of normally developed larvae (referred as to P0; Fig. 3A–B) but also contained smaller numbers of larvae with developmental defects that were further classified according to the phenotypic strength (P1 and P2, respectively; Fig. 3C–F), in addition to eggs that showed no obvious sign of development (n.d.; not shown). Differences in the proportions of the observed phenotypes between NSW and DMSO controls appeared to be non-significant (p>0.1), thus embryos incubated in 0.5% DMSO were employed as control (Fig. 3G). At the lowest U0126 inhibitor concentration tested (conditions under which MAPK activation was abrogated, Fig. S1G), we observed a significant decrease (p<0.05) of normally developed larvae (Fig. 3G) at the expense of P1-animals. These larvae were characterized by a shortened overall morphology, reduced parapodia and cilliary bands as well as smaller heads (Fig. 3C–D). Upon closer inspection, their ventral nervous system appeared less condensed, connectives were misplaced and disorganized and the commissures were shortened and irregularly formed along the body axis (Fig. 3C, C′, C″′ compare to untreated embryos in 3A–A″′). These larvae also exhibit a grossly disorganized muscle pattern with missing or not properly positioned muscles (Fig. 3C′, C″). Notably, *Pdu-Mhc* is strongly expressed in both normally developed and P1 larvae (Fig. 3B, D), suggesting that specification of the general muscle fate is not impaired as a result of the U0126 treatment. When compared with the control group, the increase of P1 larvae after 10 µM U0126 treatment was highly significant (p<0.01; Fig. 3G). Treatments with higher inhibitor concentrations (25 µM and 50 µM) did not lead to a further significant increase in the amount of P1 larvae (both p>0.1; Fig. 3G) although we observed slightly increased numbers of P2 animals (Fig. 3E–F) and not developed (n.d.) eggs. However, their proportion was not significantly increased (p>0.1) compared to the control and 10 µM treatment groups (Fig. 3G). Staining of P2 larvae with FITC-coupled phalloidin and antibodies against acetylated tubulin (aat) revealed a radialized larval morphology with no signs of muscle pattern and nervous system formation (Fig. 3E–E″′). *Pdu-Mhc* expression uncovers differentiation of muscle tissue in P2 animals but muscle cells appear reduced and form loose accumulations on the radialized larvae suggesting the complete absence of muscle pattern formation (Fig. 3F). This finding could be relevant for further studies involving U0126 treatment since radialization of treated larvae has been described as specific effect after MEK inhibition [10]. Since these observations indicated a function for MAPK signaling in the rearrangement of embryonic tissues rather than in mesodermal cell specification we analyzed control and U0126-treated embryos at the gastrulation stage (24 hpf) employing *in situ* hybridization against *Pdu-twist*. At this time point, *Pdu-twist* is expressed in three bilateral domains along the anterior-posterior axis ([8]; Fig. 3H). After treatment with 10 µM U0126 between 13.5 hpf and 16.5 hpf we observed an increased number of larvae (161 of 270 compared to 45 of 310 in the control) in which *Pdu-twist* positive cells were internalized but accumulated in the posterior region and failed to arrange in their designated positions (Fig. 3I), supporting our assumption that MEK inhibition leads to spreading defects of the gastrulating trunk mesoderm.

Taken together, our results suggest that MAPK activation does not impair the general specification of muscle and nerve cell fates. However, MAPK signaling seems to be required during gastrulation process which appears to be a prerequisite for the subsequent formation of a regular muscle pattern and a properly organized ventral nervous system.

## Discussion

The selective distribution of morphogenetic determinants to particular blastomeres has been described as a key process in early development in several species with spiral cleavage [29]–[33]. Although the existence of maternally derived morphogenetic determinants which are crucial for the early development in *Platynereis* has long been assumed [5], [7], very little is known about their molecular character and function. Recently, this assumption has been strengthened by the observation that transcripts of the potential germ cell determinant *Pdu-vasa* and the *Platynereis* estradiol receptor homolog are already present in the unfertilized egg and selectively distributed during early cleavages [21], [34]. In this study, we report that transcripts of the *twist* ortholog are also maternally contributed to the oocyte in *Platynereis dumerilii*, unveiling another factor with the potential to function as an early morphogenetic determinant. Maternal expression of *twist* has been reported in leech [33] and functional studies in the ascidian *Halocynthia roretzi* reveal a crucial role for a maternal factor, *macho-1*, in early muscle cell fate specification [35]. Therefore, an early specification of mesodermal or myogenic lineages might be a conserved feature in embryos that undergo mosaic development. We further observed that ooplasmatic segregation leads to the accumulation of *Pdu-twist* transcripts in blastomeres at the animal pole, which are further assigned into the 2d and 4d lineages by the characteristic unequal spiralian cleavage. In *Platynereis dumerilii*, 4d is referred to as the mesentoblast and former lineage tracing analysis identified this cell as progenitor of the trunk and pharyngeal mesoderm [9]. Therefore, an accumulation of *Pdu-twist* in this particular lineage might confirm its essential role as mesoderm-forming blastomere. However, our results do not reveal obvious differences in maternal *Pdu-twist* distribution between the early 4d and somatoblast (2d) lineages, with the latter giving rise to the bilateral trunk ectoderm [9]. This observation suggests that maternal *Pdu-twist* is no defining characteristic for the mesentoblast and not sufficient to specify mesodermal aspects of the 4d lineage. However, the maternal component of *Pdu-twist* could represent an important prerequisite for the later specification of the mesoderm. Therefore, it is likely that not only maternal *Pdu-twist* but also the general cytoplasmatic content of the blastomeres, their size, cell cycle, relative position within the embryo as well as cell-cell signaling events could be crucial for further specification manifested by distinct gene expression patterns. For instance, Schneider and Bowerman (2007) identified a conditional cell-fate specification mode mediated by subcellular asymmetries of β-catenin distribution. Interestingly, asymmetric β-catenin distribution is omitted in the transverse divisions of a 2d descendant and 4d, revealing their prominent roles as inducers of bilateral symmetry in the trunk. Later on, high levels of β-catenin are present in the progenitor cells of the trunk mesoderm which is remarkably reminiscent of a highly conserved aspect of mesoderm specification that involves alteration and maintenance of Twist expression levels [17], [36].

Interestingly, our results reveal a strong increase in *Pdu-twist* expression levels that can be observed in two cells that arise from the 4d lineage whereas the remaining cells appertaining to the D-quadrant contain lower levels of maternal *Pdu-twist*. Considering the time point and the results obtained from our RT-PCR analysis, it might be plausible that this reflects zygotic *Pdu-twist* expression. With respect to their location in the embryo and in accordance with a recent lineage analysis by Fischer and Arendt (2013) these high *Pdu-twist* expressing cells most likely correspond to the myogenic precursors that give rise to the future trunk musculature of the larva [19]. This is also supported by the cell tracking experiments of Ackermann et al. (2005) who identified 4d as the precursor of the trunk musculature, and our recent finding that *Pdu-twist* is strongly expressed in the trunk mesoderm during larval muscle formation [8]. Another reason why an increase in *Pdu-twist* expression is not detectable in 4d and its earliest descendants might be the separation of the primordial germ cells (PGCs) from the mesentoblast lineage [17], [19], [21]. The origin of the germ line from the mesendodermal lineage has been described for many spiralian taxa and PGC-separation is completed before this lineage specifies mesodermal cells [21], [37]–[44]. Since germ line specification is associated with a general inhibition of mRNA transcription in various taxa [45] it might be conceivable that separation of the germ line leads to the segregation of such repressive factors, thus facilitating the onset of mesoderm-specific gene expression in the sister cells.

Previous studies on molluscs revealed a close correlation between the specification of the 4d lineage and the activity of an embryonic organizer linked by activation of the highly conserved mitogen-activated protein kinase/extracellular signal-regulated kinase (MAPK/ERK) pathway [10], [11], [13], [14]. Detection of MAPK activation has further been employed to reveal the existence of an embryonic organizer in the polychaete species *Hydroides hexagonus* and *Capitella* sp. I. Surprisingly, MAPK is di-phosphorylated in the 4d blastomere of *Hydroides* but not active during early embryogenesis in *Capitella* suggesting different modes of organizer formation among these species [14], [15]. Analyzing MAPK activation in *Platynereis*, we neither detected dpERK signals in the somatoblast (2d), the mesentoblast (4d) nor their early descendants, which would be more reminiscent of early development in *Capitella*. Another striking similarity between both species is the activation of MAPK signaling within several cells at the blastopore lip [15] (this work). In *Platynereis*, the blastopore forms at the “vegetal cross furrow” between the macromeres 4D and 4B. This is also the region where the descendants of the 4d lineage submerge under the surface of the embryo [9]. By blocking MAPK activation during gastrulation in *Platynereis*, we found a significantly increased number of larvae with muscle pattern defects and failures in nervous system condensation. These defects remarkably resemble phenotypes observed in *Drosophila heartless* (*htl*) mutants [46]–[49]. *htl* encodes for one *Drosophila* FGFR homolog and is expressed in the developing mesoderm where FGF signaling, induced by the ectodermally expressed ligands Pyramus and Thisbe, is required for mesodermal cell migration and spreading [50]–[53]. Loss of *htl* function further affects the induction of mesodermal lineages particularly in the dorsal parts of the embryo [46]. Notably, MAPK di-phosphorylation is detectable in the dorsal-most mesodermal cell rows during gastrulation in *Drosophila* [54], [55], and Ras/MAPK activation downstream of FGFR-signaling has been reported to be required but not sufficient for mesodermal cell spreading [56]. Our morphological analysis reveals accumulation of actin as well as cell morphology changes in dpERK positive cells, thus exhibiting characteristics also observed in gastrulating cells. Moreover, blocking MAPK activation impairs the spreading of mesodermal progenitor cells within the gastrula which finally results in a disorganized larval muscle pattern. Comparable phenotypes were described after U0126 treatment of *Capitella* sp. I embryos. Amiel et al. (2013) also report that ablation of the 2d blastomere disrupts bilateral symmetry, dorso-ventral axis formation and impairs proper trunk mesoderm formation [15]. Since 2d is the progenitor of the bilateral trunk ectoderm it could be possible that, in absence of this tissue, the developing mesoderm lacks inductive cues which are essential for its proper positioning and organization.

But why are the temporal MAPK activation pattern and the formation of the embryonic organizer so remarkably different among the analyzed polychaete taxa? One important point is that *Capitella* as well as *Platynereis* undergo an unequal spiral cleavage whereas *Hydroides* exhibits sinistral equal cleavage [57]. Unequal cleavage allows the prediction of the future dorsal side and the secondary body axis already at the four-cell stage [1], [7], [42]. Assuming equivalence among the first four blastomeres in *Hydroides*, it could be possible that cell-cell signaling and subsequent activation of MAPK are employed later in development to induce 4d as the embryonic organizer in the D-quadrant that further defines the secondary body axis.

Taken together, our analysis unveiled a maternal contribution of *twist* in *Platynereis dumerilii* that is selectively distributed to certain cell lineages. Albeit not a particular feature of the mesentoblast lineage, maternal *Pdu-twist* could represent an important prerequisite for the later mesoderm specification which is indicated by a strong zygotic expression of *Pdu-twist* exclusively observed in the myogenic progenitors after the 4d stage. Our experiments did not reveal the existence of a conserved connection between the 4d lineage and MAPK signaling previously associated with the activity of the spiralian organizer. However, a role for MAPK signaling is revealed during the gastrulation process in *Platynereis*, where it is important for proper mesoderm spreading and tissue rearrangement.

## Supporting Information

Figure S1 **A, B.** *In situ* hybridization with a *Pdu-twist* sense probe reveals no detectable signals in early embryos. **C–D′.** Secondary antibody control staining in mid-blastula (10.5 hpf) and 15 hpf embryo. **E, F.** Fluorescein tyramide substrate precipitation within the nephroblast cells (n) at 10.5 hpf and in the head kidneys (hk). **G.** Absence of MAPK activity (dpERK) after 90 min MEK inhibition with 10 µM U0126. Scale bars: 50 µm.
(TIF)
