# Supplementary figures and images for: Maternal Inheritance of Twist and Analysis of MAPK Activation in Embryos of the Polychaete Annelid Platynereis dumerilii
Source: PLoS One. 2014 May 2;9(5):e96702. doi: 10.1371/journal.pone.0096702 (PMC4008618; doi:10.1371/journal.pone.0096702)

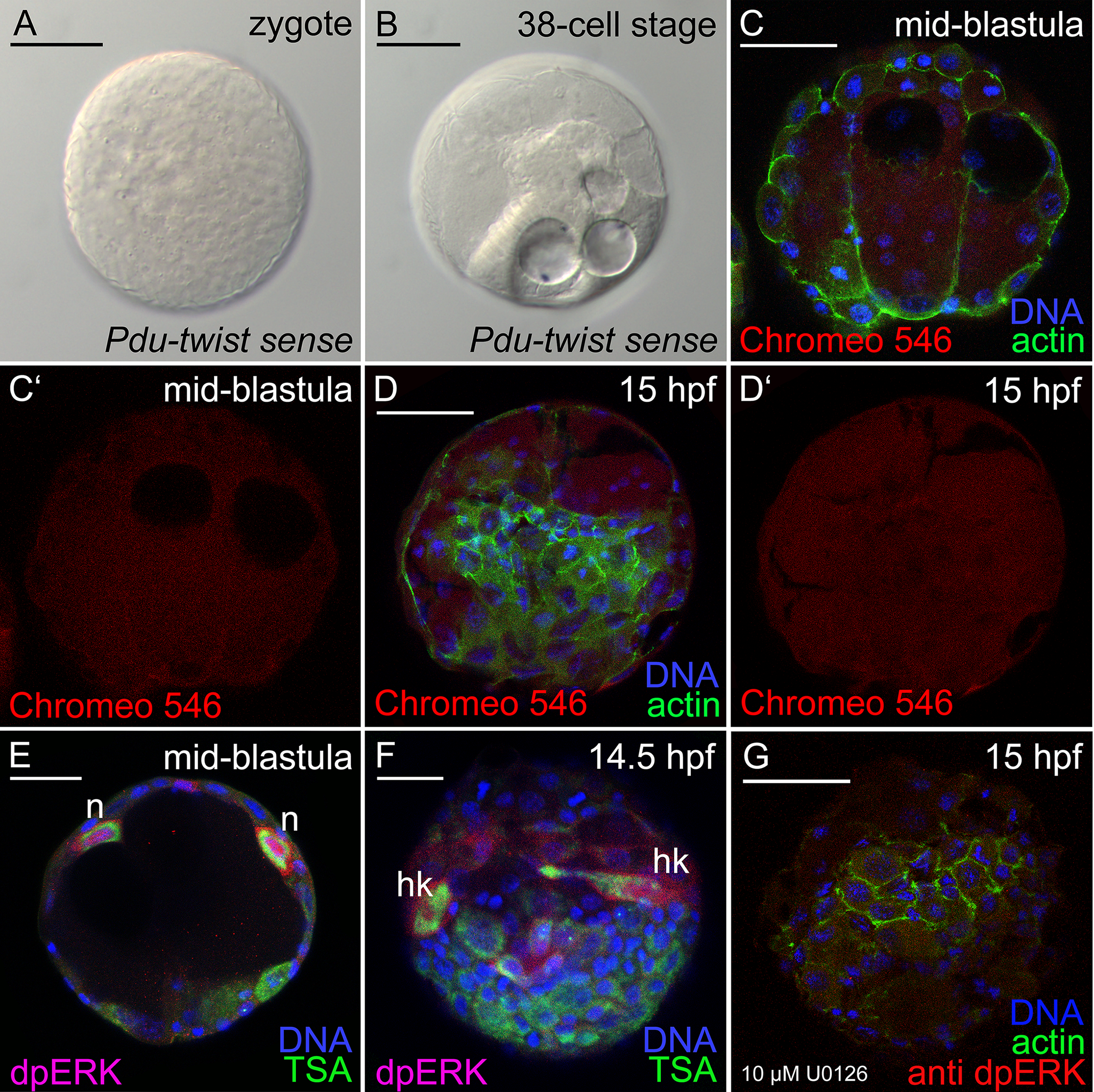

Supplement: Figure S1 — A, B. In situ hybridization with a Pdu-twist sense probe reveals no detectable signals in early embryos. C–D′. Secondary antibody control staining in mid-blastula (10.5 hpf) and 15 hpf embryo. E, F. Fluorescein tyramide substrate precipitation within the nephroblast cells (n) at 10.5 hpf and in the head kidneys (hk). G. Absence of MAPK activity (dpERK) after 90 min MEK inhibition with 10 µM U0126. Scale bars: 50 µm. (TIF) [file pone.0096702.s001.tif]
